# Amalgam

**Published:** 1858-06

**Authors:** Elisha Townsend

**Affiliations:** No. 1606 Locust st.


### AMALGAM.

Messrs. Editors:—I promised to report to you any change in my practice in the use of amalgam for filling teeth, founded upon further experience. In all that I have ever said or written upon amalgam, I have been very careful not to advocate its use except in those cases which could not be filled with gold, and where extraction was the only alternative.

I find my name has been used as authority for its indiscriminate and unlimited use, which I certainly never intended or supposed could happen.

I wish now to say to the profession, that I have entirely abandoned it, and shall never use it again in my practice. I have come to this resolution for reasons which I will now state.

In many of the cases where I most relied upon it, and expected to have the best results, it has entirely failed; as in the buccal cavities in molars, when they extended beneath the free margin of the gum, I found that while in some mouths the material remained white and clean, in others it became very black in a few days, and in almost all cases, upon removing the filling, the under side was blackened, and the same color given to the tooth. Again, in the infirm teeth for which it seemed the only thing, and for which it was best adapted by its plastic nature, many of them have had to be removed, owing to suppuration of the gums, caused by the tight closing of the previous vent for the escape of pus.

Therefore, I have come to this broad conclusion, that a tooth so infirm as to need a soft filling, would be best removed, for the health of the mouth and the health of the patient; and that my practice hereafter will be to advise their removal, and then leave the responsibility with the patient.

ELISHA TOWNSEND,

No. 1606 Locust st.

—*Dental News Letter.*

